# Sodium‐glucose cotransporter‐2 inhibitor initiation and incident dementia in heart failure with diabetes: a population‐based cohort study

**DOI:** 10.1002/alz70859_096860

**Published:** 2025-12-25

**Authors:** Che‐Yuan Wu, Abhinav Sharma, Jodi D. Edwards, Peter P. Liu, C Fangyun Wu, Sho Podolsky, Moira K. Kapral, Krista L Lanctôt, Bradley J. MacIntosh, Hugo Cogo‐Moreira, Baiju R. Shah, Walter Swardfager

**Affiliations:** ^1^ Sunnybrook Research Institute, Toronto, ON Canada; ^2^ University of Toronto, Toronto, ON Canada; ^3^ McGill University, Montreal, QC Canada; ^4^ University of Ottawa, Ottawa, ON Canada; ^5^ University of Ottawa Heart Institute, Ottawa, ON Canada; ^6^ ICES, Toronto, ON Canada; ^7^ Toronto General Hospital Research Institute, University Health Network, Toronto, ON Canada; ^8^ KITE Toronto Rehabilitation Institute, Toronto, ON Canada; ^9^ Ostfold University College, Halden Norway; ^10^ Sunnybrook Health Sciences Centre, Toronto, ON Canada

## Abstract

**Background:**

People with heart failure have an increased risk of developing dementia. Commonly comorbid diseases such as diabetes, atrial fibrillation, and hypertension can further elevate dementia risk. However, people with heart failure are underrepresented in clinical trials for dementia. This study aims to determine whether sodium‐glucose cotransporter‐2 inhibitors (SGLT2i) use is associated with a lower risk of dementia in people with heart failure and comorbid diabetes.

**Method:**

This study used linkable administrative databases from Ontario, Canada housed at ICES. Dementia‐free individuals aged ≥66 years with heart failure and diabetes who initiated an SGLT2i or dipeptidyl peptidase‐4 inhibitor (DPP4i), from July 1^st^, 2016 to December 31^st^, 2020, entered this cohort. DPP4i was selected as an active comparator to reduce confounding by indication. A 180‐day lag time was implemented to mitigate reverse causation. The primary analysis used an intention‐to‐treat exposure definition. Propensity score fine stratification weights were used to adjust for baseline covariates, including demographics, comorbidities, procedures, medications, healthcare utilization, and disease‐related variables. The definition of dementia was adapted from previously validated algorithms. Cause‐specific hazard ratios (HRs) with death as a competing risk were estimated, using weighted Cox regression models.

**Result:**

Among 4,402 SGLT2i and 6,319 DPP4i new users, the mean age was 76.4 years, and 39.7% were female. Over a median follow‐up of 3.95 years from cohort entry, SGLT2i versus DPP4i initiation was associated with a 26% lower risk of incident dementia (HR 0.74, 95% confidence interval 0.61‐0.89; incidence rate: 21.8 versus 29.5 events per 1,000 person‐years). Associations between SGLT2i initiation and a lower dementia risk persisted in subgroups with atrial fibrillation (0.66, 0.51‐0.85; 20.6 versus 31.2) or those aged ≥75 years (0.62, 0.50‐0.78; 26.9 versus 43.1). The secondary as‐treated analysis showed greater dementia risk reduction (0.54, 0.40‐0.73; 16.3 versus 30.1).

**Conclusion:**

SGLT2i initiation was associated with a lower risk of dementia in people with heart failure and diabetes, particularly in high‐risk subgroups for dementia. The findings suggest that SGLT2i may prevent dementia in these high‐risk populations, representing new opportunities for randomized controlled trials.

